# Mitral annular calcification in patients with significant mitral valve disease: An old problem with new solutions

**DOI:** 10.3389/fcvm.2022.1033565

**Published:** 2022-11-21

**Authors:** Guido Ascione, Paolo Denti

**Affiliations:** Department of Cardiac Surgery, Istituto di Ricovero e Cura a Carattere Scientifico (IRCCS) San Raffaele Hospital, Vita-Salute San Raffaele University, Milan, Italy

**Keywords:** mitral valve regurgitation, mitral annular calcification (MAC), Tendyne, TMVR in MAC, MAC quantification

## Abstract

Mitral annular calcification (MAC) is a chronic process involving mitral valve annulus, linked with an increased cardiovascular mortality and morbidity. Since its first autoptic description, a progressive evolution in diagnostic tools brought cardiac computed tomography (CT) scan to become the gold standard in MAC detection and classification. The treatment of significant mitral valve disease in patients with annular calcifications has always represented an issue for cardiac surgeons, being it linked with an increased risk of atrioventricular groove rupture, circumflex artery injury, or embolism. As a consequence, different surgical techniques have been developed over time in order to reduce the incidence of these fearsome complications. Recently, transcatheter mitral valve replacement (TMVR) has emerged as a valid alternative to surgery in high-risk patients. Both hybrid transatrial, transfemoral, or transapical approaches have been described to deliver balloon-expandable or self-expanding aortic transcatheter valves into the calcified annulus, with conflicting early and long-term results. Tendyne (Abbott Structural Heart, Santa Clara, CA, USA) is a promising transapical-delivered option. Early results have shown effectiveness and safety of this device in patients with MAC and severe mitral valve disease, with the lowest rate of embolization, mortality, and left ventricular outflow tract obstruction (LVOTO) reported so far.

## Introduction

Mitral annular calcification (MAC) is a chronic process characterized by a progressive calcium deposition at the level of mitral annulus. Its prevalence is estimated between 8 and 15% (1), but it significantly increases with age and has been especially associated with altered calcium metabolism, for example in patients with chronic kidney disease.

The presence of MAC itself has been linked with an increased cardiovascular mortality and morbidity (2). Moreover, the coexistence of MAC and significant mitral regurgitation or stenosis has historically represented a challenge for cardiac surgeons, being mitral valve interventions in this context associated with an increased risk of cardiac rupture at the atrioventricular junction, perivalvular leaks, circumflex artery injury, and embolism (1). For these reasons, patients with severe annular calcifications are often deemed too high risk to undergo surgery. On the other hand, according to a recent report (3), subjects with MAC and significant mitral disease, if left untreated, show poor outcomes. These may be improved with either surgical or transcatheter interventions.

Aim of this paper is therefore to describe MAC and its classification and review all the available approaches to treat coexistent significant mitral disease: surgical treatment, transatrial hybrid procedures, and percutaneous treatment.

## Mitral annular calcification and its diagnosis

Mitral annulus is a complex saddle-shaped structure separating left atrium and left ventricle. Anteriorly, it is in close continuity with aortic root and aorto-mitral curtain. Posteriorly, the fibrous layer is discontinuous and periodically interrupted by fat tissue (1).

Calcifications involving mitral annulus have been already described in the early nineties in autoptic studies (4), but the first comprehensive evaluations came later in the context of surgical series.

Carpentier et al. (5), specifically, analyzed 68 patients with MAC referred in a 10-years span (1986–1995) to surgery for concomitant mitral regurgitation. As a result of a broad assessment, based on both pre-operative and intra-operative findings, calcifications were described as involving at least one third of the posterior annulus in 88% of the cases, the whole posterior annulus in 10% and also the attachment of the anterior leaflet in 1.5% of the cases. Furthermore, the degenerative process was limited to the annulus itself in most of the patients, while extra-annular structures were interested by calcifications in 25% of the subjects (12% ventricular wall, 6% posterior leaflet, and 4.5% papillary muscles). Interestingly, MAC was found to be usually coated by a fibrous sheath, so that calcifications are basically separated from the surrounding structures. This distinction is not well demarcated where the degenerative process infiltrates left ventricular myocardium.

Both chest X-ray and coronary angiography may reveal annular calcifications as a C or O-shaped ring lying at left atrioventricular junction, but they cannot help in defining the extension of the degenerative process (1).

**FIGURE 1:**
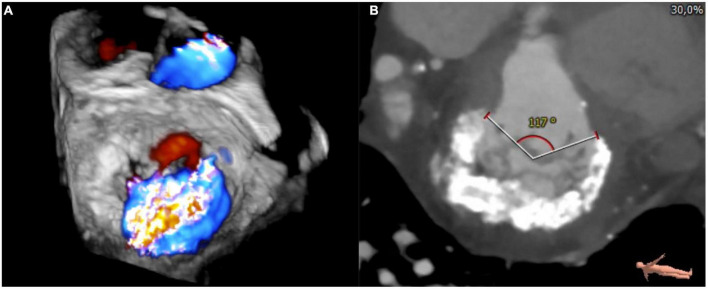
3D transesophageal echocardiography **(A)** and cardiac computed tomography (CT)-scan **(B)** showing severe mitral annular calcification (MAC) and coexisting severe mitral regurgitation.

Ecocardiography had been considered for a long time the best tool to detect MAC (Figure 1). Annular calcifications are visible, using M-mode, as a dense echo band lying below the posterior mitral leaflet, with a motion pattern paralleling that of free ventricular wall (6). Two-dimensional echo, on the other hand, is useful to define MAC morphology. With this ultrasound modality, calcifications appear as highly reflective irregular structures at the junction between atrioventricular groove and posterior mitral leaflet, with associated acoustic shadowing (1). Different echocardiographic methods to define MAC severity have been described. Barash et al. (7) proposed a qualitative classification, based on parasternal long axis view projections. MAC was defined as “mild” in presence of only focal calcifications, confined to mitral annulus, “moderate” when more than 1/3 but less than 1/2 of mitral annulus is involved and “severe” when more than half of ring circumference is affected, with calcifications intrusion into left ventricular wall. A subsequent quantitative classification (8), based on MAC maximal thickness when measured at its greatest width, defined MAC as severe when a value > 4 mm is recorded.

With the diffusion of cardiac computed tomography (CT), ECG-gated methods rapidly took over echocardiography as gold standard in MAC diagnosis and quantification (Figure 1). Cardiac CT scan shows a higher spatial resolution in distinguishing heart structures, allowing a better identification of calcifications exact location (1). As a consequence, it has been used to develop MAC quantification scores.

Guerrero et al. (9), through a retrospective analysis of 87 baseline cardiac CT scan of Valve-in-MAC candidates, proposed a scoring system of MAC severity.

**FIGURE 2:**
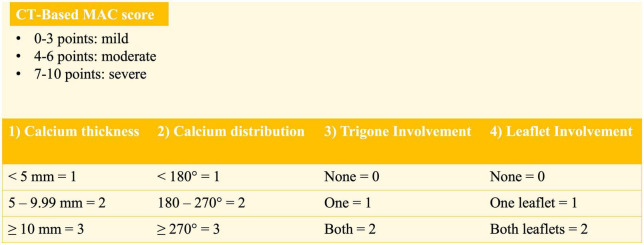
Computed tomography (CT)-based mitral annular calcification (MAC) score according to Guerrero et al. (9).

Four characteristics are taken into account: calcium thickness, calcium distribution in the annulus circumference, calcification of one or both fibrous trigones, and leaflet involvement. The sum of points acquired in each of these categories makes the final score, with a score of 3 or less representing mild MAC, 4 to 6 moderate MAC and 7 or more severe MAC (Figure 2).

The authors used this score to predict the risk of valve embolization/migration after transcatheter mitral valve replacement (TMVR) using aortic transcatheter heart valves. Embolization/migration rates were lower in higher scores, with a score of 6 or less identified as an independent predictor of valve embolization/migration.

## Treatment options

Patients with MAC and significant mitral valve disease represent a high-risk surgical population. In fact, mitral surgery in this context is linked to an increased threat of atrioventricular junction rupture, circumflex artery injury, and embolism (1). As a consequence, alternative treatments have been developed over time. At first, transcatheter aortic valve prosthesis delivery inside the calcified annulus has been proposed as an option, with both open access to left atrium, a transfemoral, or a transapical approach.

New devices, specifically designed to fit the complex shape of mitral annulus, have been lately developed in order to treat mitral valve disease in patients deemed too high risk to undergo conventional surgery. Among them, Tendyne system showed very interesting results in MAC population.

### Surgical treatment

**FIGURE 3:**
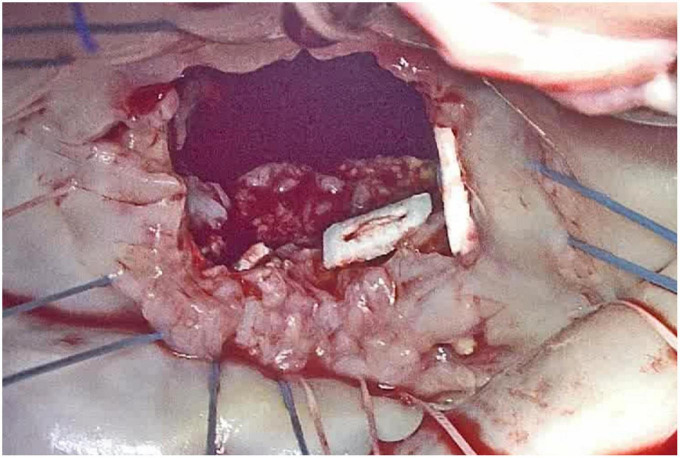
Intra-operative appearance of mitral annular calcification (MAC) during surgical inspection, after anterior mitral leaflet removal.

Surgical mitral valve repair or replacement remain the gold standard to treat patients with mitral valve pathology, even in presence of severe MAC (Figure 3).

Historically, two possible approaches have been depicted: either extensive annular decalcification and reconstruction (“resect” strategy) or a more conservative approach, that minimizes the risks linked with calcium removal (10).

Several “resect” strategies have been described.

Carpentier et al. (5) pioneered the en-bloc removal of posterior annular calcifications, from one trigone to the other, by sharp dissection. After atrial endocardium incision and posterior leaflet detachment to expose both atrial and ventricular side of MAC, the calcium bar is removed with its fibrous sheath. Annular reconstruction is then performed either by interrupted sutures between atrial and ventricular annular edges or, if decalcification reaches the ventricular myocardium, with the so called “sliding atrium technique.” It consists of atrial annular edge dissection to create an atrial flap, which is then mobilized and used to cover the decalcified area. Following annular reconstruction, mitral valve repair or replacement is then performed.

Results of this aggressive approach in 67 patients (5) showed an in-hospital mortality rate of 3.3%, a 7-years survival of 93.1%, a freedom from reoperation for mitral regurgitation at 9 years of 87.1% and significant valvular leaks at follow-up in about 10% of the patients.

David et al. (11) described a similar extensive decalcification approach, but annular reconstruction was achieved using autologous pericardium. However, the long-term results of this aggressive strategy were significantly worse in their series, with a survival rate of 49% at 8 years.

More conservative approaches include partial decalcification or MAC avoidance instead of removal, with suture placement around the calcium bar, both behind it or on mitral leaflets (10).

More recent results about mitral valve surgery in patients with MAC showed an operative mortality between 1 and 5.8% and a survival rate at 5 years between 38.8 and 78.8% (10).

### Transatrial hybrid procedure

Looking for alternatives to conventional mitral valve surgery, one of the first options to be explored was the direct delivery of a balloon expandable transcatheter valve inside the calcified annulus, using a hybrid strategy that involves cardiopulmonary bypass, cardioplegic arrest and surgical left atriotomy. The first successful implantation was achieved in 2012 (12).

Different types of transcatheter aortic valves were used, even if most of the patients received SapienXT prosthesis or Sapien3 (Edwards Lifesciences, Irvine, CA, USA) (13). Before valve delivery, anterior mitral leaflet is usually resected and thus transatrial access offers a very low risk of left ventricular outflow tract obstruction (LVOTO). Moreover, a felt strip is typically sutured around the inflow of the valve and, through pledgeted stiches, directly onto valve leaflets remnants. These steps help to reduce the risk of valve embolization and perivalvular leaks (14).

The biggest experience reported so far, on a sample of 26 patients with severe MAC (14), revealed a procedural success in 100% of patients. However, both in-hospital and 30-days mortality were high (20 and 27%, respectively).

This unsatisfactory survival results were confirmed also by a prospective trial (MITRAL), that showed an in-hospital, 30-days, and 1-year mortality of 9.5, 20.0, and 40%, respectively (13).

### Percutaneous treatment (transfemoral, transapical)

Since the beginning of TMVR experience, other delivery strategies were explored, alternatively to direct implantation through left atriotomy. These approaches, differently from the hybrid procedure, are performed on a beating heart, without cardiopulmonary bypass assistance.

Both transfemoral and transapical TMVR have been described.

In the first case, the delivery system is advanced through the femoral vein until the right atrium, where a transeptal puncture allows access to the left atrium. The transapical approach, on the other hand, requires a left anterior thoracotomy, and mitral valve is then reached from its ventricular side.

Most of the available reports about these approaches are retrospective and include a small cohort of patients. The most used valves were SapienXT prosthesis or Sapien3 (Edwards Lifesciences, Irvine, CA, USA) (13).

Data collected so far showed a variable technical success (between 62 and 92%), with an embolization rate ranging from 0 to 16.7%. LVOTO occurred in 10 up to 39.7% of the cases. The reported 30-days mortality ranged from 11.1 to 34.5% (13).

The only available prospective study (MITRAL) (13), who enrolled 100 patients, substantially confirmed what previously stated, with a technical success in 68.8% of the cases, LVOTO in 13.4% of the cases and a 30-days mortality of 13.4%.

A recent systematic review (13) calculated a median incidence of at least moderate post-procedural mitral regurgitation of 4.1%. Overall, the median in-hospital, 30-days, and 1-year mortality rates for non-transatrial TMVR in MAC were 16.7, 22.7, and 43%, respectively.

### Tendyne

Tendyne (Abbott Structural Heart, Santa Clara, CA, USA) is a self-expanding, repositionable nitinol prothesis that is delivered *via* a transapical sheath and anchored at cardiac apex with a tether connected to an epicardial hemostatic pad (15). It has been specifically designed to fit the complex 3-dimensional shape of mitral annulus [lower occurrence of paravalvular leaks (PVL)], and, thanks to its anchoring pad, to reduce the risk of embolization.

The Global Feasibility Study (30 subjects enrolled) showed safety and efficacy of this device in treating patients with significant mitral regurgitation, deemed too high-risk to undergo conventional surgery (15).

**FIGURE 4:**
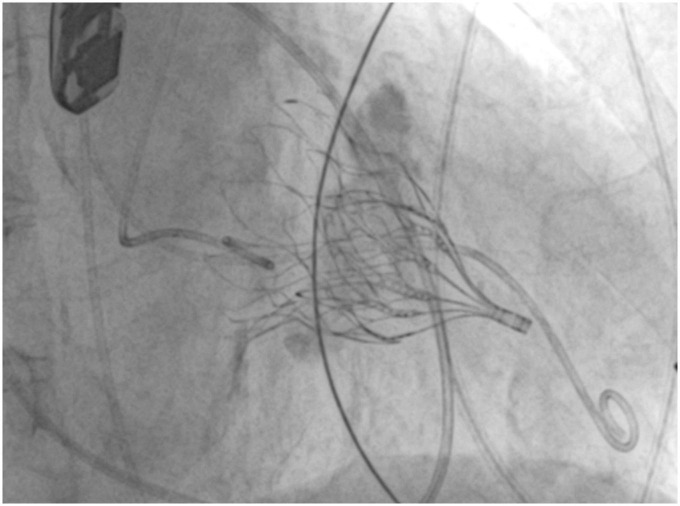
Fluoroscopy image showing a successful Tendyne implantation in severe mitral annular calcification (MAC).

Gössl et al. (16) recently published early outcomes of TMVR with Tendyne in patients with severe MAC (Figure 4). Among 20 enrolled patients (9 compassionate use, 11 taken from the Feasibility Study of Tendyne in MAC), both acute and midterm outcomes were encouraging. In fact, 30-days all-cause mortality and 1-year cardiac mortality were 5 and 20%, respectively, with no recurrence of mitral regurgitation and clinical improvement in 92% of patients who were alive at 1-year follow-up.

The SUMMIT trial (NCT03433274), still ongoing, is the pivotal clinical trial testing feasibility and safety of the Tendyne device in the United States. The primary endpoint is survival free of heart failure hospitalization at 12 months (15). Among the 3 available cohorts, one is dedicated to evaluate results in the subgroup of patients with severe MAC. With more than one hundred enrolled subjects in this cohort so far, this prospective study will help to understand what’s the role Tendyne valve may have in treating patients with significant mitral regurgitation and severe annular calcifications.

## Discussion

The presence of MAC in patients with significant mitral valve disease represents a challenging anatomical scenario, both from a surgical and a transcatheter point of view. This complexity has fostered the introduction of different treatment options.

Conventional surgery still remains the preferred intervention in patients with acceptable surgical risk. However, both extensive annular decalcification or conservative, calcium-respectful, approaches are linked with an increased risk of atrioventricular groove rupture, and circumflex artery injury. As a consequence, even if the results of mitral valve repair in terms of recurrence of significant MR are positive (freedom from reoperation 87% at 9 years) (5), reported mortality rates are still high (operative mortality between 1 and 5.8% and a survival rate at 5 years between 38.8 and 78.8%) (10).

It must be considered, however, that most of the available surgical series are old and small in size. A recent report (17), retrospectively analyzing 9,551 patients with MAC undergoing mitral valve surgery, has on the other hand the big limitation of not assessing long term outcomes.

The introduction of TMVR options has widened the armamentarium available to treat this complex population. Differently from surgical mitral valve repair/replacement, transcatheter delivery is linked with different intra-procedural threats, namely PVL, valve migration, and LVOTO.

Even if the first two complications were more frequent at the beginning of TMVR experience, better patients’ and device selection significantly reduced their incidence. In fact, both PVL and embolization usually resulted from device undersizing, or insufficient MAC to ensure adequate valve anchoring (13).

Unlike in surgical mitral valve replacement, anterior mitral leaflet cannot be removed during TMVR, thus increasing the risk of LVOTO.

In a recent systematic review (13), the median incidence of LVOTO in transatrial, transfemoral, and transapical TMVR (not including Tendyne) was 13.4%. Different strategies have been developed to prevent obstruction, including alcohol septal ablation (both precautionary or as a bailout), intraoperative resection of the anterior mitral leaflet and septal myectomy during transatrial implantation and the LAMPOON (Laceration of the Anterior Mitral Leaflet to Prevent LVOTO) approach (18).

The latter, albeit technically complex, showed a procedural success of 100% and was able to reduce LVOT gradient to less than 30 mmHg in 97% of patients in a retrospective study on TMVR in MAC (19).

As a matter of fact, even if linked with a low rate of LVOTO (less than 10% in the prospective trial MITRAL) (13), transatrial hybrid TMVR remains a surgical operation, with a non-negligible mortality (20% at 30 days, 40% at 1 year) and an in-hospital major bleeding rate ranging from 6.7 to 25% (13).

Tranfemoral and transapical approaches, on the other hand, showed comparable survival rates (median 30-days mortality 22.7%, median 1-year mortality 43%), with a risk up to 16.7% of valve embolization (20), and an LVOTO rate ranging from 7.4 to 39.4% (13).

In this scenario, Tendyne represents a promising alternative.

In fact, the early available results (16) show a 95% technical success, with a 30-days mortality rate of 5% and a 1-year cardiac mortality of 20%. Only one patient of the cohort developed LVOTO, successfully treated by septal ablation.

However, these encouraging outcomes are, to some extent, the result of a very selective patients’ recruitment process, with a screen failure rate due to unfavorable anatomy of at least 40% (13).

Larger perspective studies are needed to confirm the available results.

## Conclusion

In the complex anatomical and clinical context of patients with MACs and significant mitral valve disease, conventional surgery still represents in eligible subjects the gold standard of treatment, capable of ensuring durable results. TMVR has emerged as an interesting alternative in high-risk patients, and the progressive technological and procedural evolution is gradually reducing the incidence of PVL, embolization, and LVOTO.

With its promising early results, Tendyne valve may set a new benchmark in transcatheter treatment of mitral valve disease in patients with annular calcifications.

Further steps include optimization of patient selection and pre-procedural planning, in order to create a standardized treatment algorithm that could offer the best solution for each patient.

## Author contributions

GA: conceptualization, data curation, investigation, visualization, and writing – original draft. PD: validation, supervision, and writing – review and editing. Both authors contributed to the article and approved the submitted version.
